# Design and development of a complex narrative intervention delivered by text messages to reduce binge drinking among socially disadvantaged men

**DOI:** 10.1186/s40814-018-0298-0

**Published:** 2018-06-06

**Authors:** Linda Irvine, Ambrose J. Melson, Brian Williams, Falko F. Sniehotta, Gerry Humphris, Iain K. Crombie

**Affiliations:** 1 Division of Population Health Sciences, University of Dundee, The Mackenzie Building, Kirsty Semple Way, Dundee, DD2 4BF UK; 2 Institute of Health and Wellbeing, University of Glasgow, Mental Health and Wellbeing Academic Centre, Gartnavel Royal Hospital, 1055 Great Western Road, Glasgow, G12 0XH UK; 3 School of Health and Social Care, Edinburgh Napier University, Sighthill Campus, Sighthill Court, Edinburgh, EH11 4BN UK; 4 Institute of Health and Society, Newcastle University, Baddiley-Clark Building, Richardson Road, Newcastle, NE2 4AX UK; 5 Medical and Biological Sciences, School of Medicine, University of St Andrews, North Haugh, St Andrews, KY16 9TF UK

**Keywords:** Behaviour change intervention, Narrative intervention, Text messages, SMS, Binge drinking, Complex intervention

## Abstract

**Background:**

Socially disadvantaged men are at high risk of suffering from alcohol-related harm. Disadvantaged groups are less likely to engage with health promotion. There is a need for interventions that reach large numbers at low cost and which promote high levels of engagement with the behaviour change process. The aim of this study was to design a theoretically and empirically based text message intervention to reduce binge drinking by socially disadvantaged men.

**Results:**

Following MRC guidance, the intervention was developed in four stages. Stage 1 developed a detailed behaviour change strategy based on existing literature and theory from several areas. These included the psychological theory that would underpin the intervention, alcohol brief interventions, text message interventions, effective behaviour change techniques, narratives in behaviour change interventions and communication theory. In addition, formative research was carried out. A logic model was developed to depict the pathways between intervention inputs, processes and outcomes for behaviour change. Stage 2 created a narrative which illustrated and modelled key steps in the strategy. Stage 3 rendered the intervention into a series of text messages and ensured that appropriate behavioural change techniques were incorporated. Stage 4 revised the messages to ensure comprehensive coverage of the behaviour change strategy and coherence of the narrative. It also piloted the intervention and made final revisions to it.

**Conclusions:**

The structured, systematic approach to design created a narrative intervention which had a strong theoretical and empirical basis. The use of a narrative helped make the intervention realistic and allowed key behaviour change techniques to be modelled by characters. The narrative was intended to promote engagement with the intervention. The intervention was rendered into a series of short text messages, and subsequent piloting showed they were acceptable in the target group. Delivery of an intervention by text message offers a low-cost, low-demand method that can reach large numbers of people. This approach provides a framework for the design of behaviour change interventions which could be used for interventions to tackle other health behaviours.

**Electronic supplementary material:**

The online version of this article (10.1186/s40814-018-0298-0) contains supplementary material, which is available to authorized users.

## Background

Socially disadvantaged men are at high risk of alcohol-related harm [1, 2]. Binge drinking (consumption of more than eight UK units (64 g) of alcohol on a single occasion) is common among young to middle-aged disadvantaged men (proportion binge drinking in the most deprived areas, 17.5 vs 10.6% in least deprived areas) [3]. It is likely to contribute to the disparity in alcohol-related harm [4]. Many alcohol interventions have been developed to tackle alcohol-related problems, and systematic reviews have shown they are effective [5–8]. However, the uptake of public health interventions among socially disadvantaged men is low [9]. Behaviour change interventions are also less effective with disadvantaged and low-income groups [10–13]. There is a need for a sensitive, tailored intervention which accesses and effectively reduces binge drinking in this hard-to-reach population. This paper reports on the systematic approach taken to the design of an intervention to tackle binge drinking in disadvantaged men. Socially disadvantaged men were identified as those living in areas of high deprivation (most disadvantaged quintile), as defined by the Scottish Index of Multiple Deprivation (SIMD) [14].

## Results

### Approach to intervention design

**Fig. 1:**
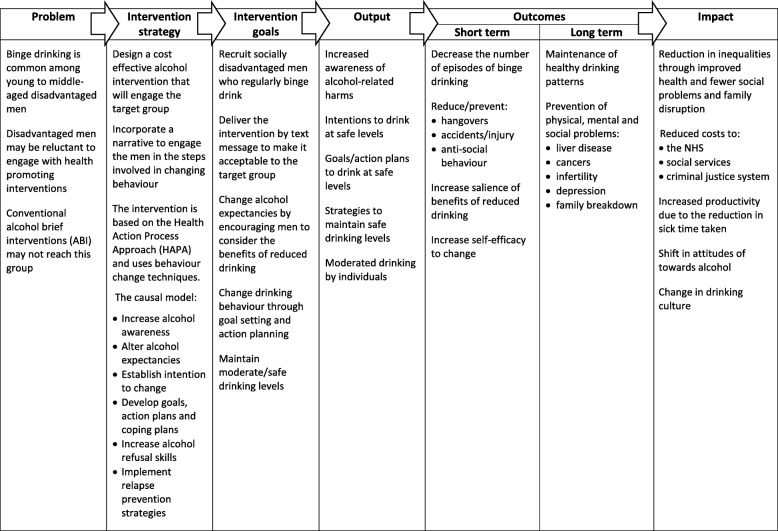
Logic model. The logic model depicts the pathways between intervention inputs, processes and outcomes for behaviour change

The importance of a systematic approach to the design of interventions is widely recognised [15–17]. This intervention was developed following the MRC framework [18] for complex interventions. As part of the process, a logic model [19] was produced to clarify the relationships between the proposed mechanisms of action, the intended outputs and longer term outcomes. The design process was conducted in four stages. The first stage involved the design of a theoretically and empirically based intervention. The starting point was a review of relevant theory and evidence, which in the present study comprised alcohol interventions and behaviour change theory and techniques. The review identified that text messages were the most suitable method for intervention delivery. This background work, together with a taxonomy of intervention features [20], informed the construction of the logic model (Fig. 1). The logic model identified several issues on which further information was required for the design of the intervention. These were explored in formative research comprising focus groups and a feasibility randomised trial [21]. Based on the findings from this stage, an initial behaviour change strategy was developed.

The next two stages involved the translation of the behaviour change strategy into a format in which it could be delivered to disadvantaged men. Stage 2 developed the narrative within which the intervention could be embedded. At this stage, narrative devices were integrated to become important elements of the behaviour change strategy. The narrative-based intervention was then rendered into a series of interactive text messages in the third stage. This stage also exploited advantages offered by delivering an intervention by text messages. At the final stage, the text messages were reviewed, piloted and revised to ensure they delivered an easily understood, coherent and comprehensive intervention. Although the description of the intervention development is presented in a linear sequence, in practice, there was considerable iteration, with decisions taken at a later stage feeding back to earlier design choices. The final set of messages is included in Additional file 1 (intervention text messages).

### Stage 1: Developing the initial behaviour change strategy

#### Reviewing brief interventions to address alcohol-related harm

The literature on alcohol brief interventions (ABIs) was reviewed to identify effective mechanisms used in previous research. ABIs are widely used, but their mechanism of action of is not clear [22–24], partly because interventions are very heterogeneous. They may contain elements of motivational interviewing (MI), feedback and advice, self-monitoring of alcohol consumption, self-help manuals, counselling and cognitive behavioural therapy [25–27]. Bien and colleagues [25] summarised the common components of effective brief interventions with the acronym FRAMES: Feedback on current consumption, Responsibility of the individual for his drinking, Advice to change, Menu of change strategies, Empathy in the delivery and Self-efficacy for action. One of the reviews has suggested that effective interventions contain at least two of three elements: feedback on drinking, advice and goal setting [28]. A more recent review found that promoting self-monitoring was the only technique that appeared effective [29]. All of these components were considered for inclusion in the intervention.

#### Outline of the psychological theory underpinning the intervention

The Health Action Process Approach (HAPA) [30] was chosen as the theoretical model to underpin the intervention because it guides participants through the behaviour change sequence, from a starting position of having no intention to change behaviour. It also addresses the intention-behaviour gap [31] identified as a weakness in some behavioural change theories. HAPA is a comprehensive model which allows integration of a range of evidence-based behaviour techniques. This model suggests the adoption, initiation and maintenance of a new health behaviour occur as a process that involves a motivational phase and a volitional phase. Motivation can be increased by altering perceived risk of a behaviour and the perceived benefits of changing that behaviour. The volitional phase includes planning, action and maintenance. Perceived self-efficacy has a crucial role in achieving success throughout the process. It helps in building an intention to change (motivation) as well as in implementing intentions into sustained action (volition). Although HAPA was used as the overarching structure, the intervention also drew on other relevant social cognition and self-regulatory models, e.g. subjective norms from the theory of planned behaviour [32] and self-monitoring from social cognitive theory [33] and control theory [34] .

#### Incorporating behaviour change techniques

Taxonomies of behaviour change techniques (BCTs) have been published to aid the design and reporting of interventions [35], including one for alcohol interventions [29]. Several techniques were incorporated into the intervention, such as providing normative information about others’ behaviour and experiences and facilitating goal setting and action planning.

#### Selection of delivery method

Given the low uptake of public health interventions among socially disadvantaged individuals [9], a key requirement of the delivery method was that it would reach and engage disadvantaged men. Text messaging provides a method for delivering brief alcohol interventions which has the potential to reach large numbers of individuals at low cost. Systematic reviews have shown that text message interventions can successfully modify adverse health behaviours [36, 37]. Mobile phone ownership in the UK is high [38] and phone users frequently check their phones [39], so study participants would be likely to open and read the messages. Previous studies report that text messages are usually read soon after delivery [40]. Texts can reach people wherever they are [41]. If a participant’s phone is switched off, messages can be accessed when the phone is switched on again. The use of text messaging may increase the salience of an intervention [42], and almost all text messages are read within minutes of delivery [40]. Text messaging is particularly suited to the target group because little effort is required to receive the intervention and texts can be accessed at times that suit the participants. In addition, each text message can be read quickly and re-read if desired. Men who may not want to commit time reading leaflets or large sections of text may prefer to receive concise text messages.

#### Logic model

From the review of the literature on alcohol brief interventions, psychological theory and text messaging, a logic model was developed (Fig. 1). This provided a detailed specification of the problem to be addressed (regular binge drinking by socially disadvantaged young to middle-aged men) and clarified what the intervention aimed to achieve. It also identified challenges to be considered, such as the lower effectiveness of health promotion interventions in disadvantaged groups [10–13]. For this study, the initial requirements for behaviour change were an increased awareness and personal relevance of the harms of alcohol and the benefits of moderated drinking. Altering risk perception and alcohol expectancies are prerequisites for increasing motivation to change. Setting goals and making action plans would lead to reduced drinking but only if self-efficacy for action had also been increased. Further, to prevent relapse, reduced drinking would have to be maintained. This could be achieved by increasing the salience of the benefits of reduced drinking and developing coping skills. Thus, the short-term benefits of moderated drinking could be used to encourage longer term reductions. This would lead to improved health and social wellbeing for the individual and a reduction in the costs of alcohol-related harms for society.

#### Formative research

The development of the text message intervention was informed by a prior feasibility study [43], which comprised focus groups and a pilot trial. The focus groups gave insight into the target group’s patterns of drinking, their knowledge about alcohol-related harms and benefits of reduced drinking.

- The common pattern of drinking was periods of abstinence interspersed with infrequent heavy drinking days, i.e. binge drinking.

I used to go out every weekend, but I’m only once a month now. That’s why I like to go out for a bucketful and that’s why I like money in my pocket to go out. (FG3, Mark, 38 years)

- Many men believed that their drinking behaviours, motives and desire to change were significantly different from when they were younger. They thought they had adopted the role of the ‘mature drinker’ which came with social roles and responsibilities (employee, husband/partner, parent). Despite this, they continued to binge drink.

With my job and responsibilities, I can’t just go out, I’m not working in a warehouse like I was when I was 18 where you can just do what you want the next day, it doesn’t matter. But now, during the week, it just can’t happen, with my job the next day, I just couldn’t function properly. (FG1, Jim, 31 years)

- Most men were aware of the harms associated with alcohol misuse but had low perceived personal risk.

You always regret it the next day, but come Friday, if you’ve not been drinking all week, and you’re feeling good again, and somebody’s offering a night out you look forward to it, you’re back on the drink.(FG1 Darren, 28 years)

- The men did not want to be preached at or told what to do in an intervention.

I always felt that nobody could dictate to you. I mean I live my life the way I see fit and I wouldn’t tell anybody else what way they should live, that’s just the way I am. (FG2, Alan, 50 years)

The subsequent randomised controlled feasibility study [43, 44] showed that:

- Participants enjoyed the interactive nature of the intervention and gave carefully considered personal responses to questions asked in the text messages
- Participants engaged with the cognitive antecedents to reducing drinking as they were discussed in the text messages

### Stage 2: The creation of a narrative

Narratives are increasingly being used as a tool for behaviour change. Hinyard and Kreuter define a narrative as ‘any cohesive and coherent story with an identifiable beginning, middle, and end that provides information about scene, characters, and conflict; raises unanswered questions or unresolved conflict; and provides resolution’ [45]. Instead of presenting facts and arguments for changing behaviour, a narrative intervention translates these into actions and experiences of characters within a chronological series of events [46]. Information presented in a narrative has a stronger effect on knowledge, attitudes and intentions than the same information in a non-narrative format [47]. Narrative is particularly useful for changing perceived social norms and behavioural intentions [48, 49]. To be effective for behaviour change, the narrative and the characters in it have to engage the reader, a process aided if the protagonists are culturally similar to the target audience [50, 51]. The narrative also has to be plausible and internally consistent [52]. The depiction of a character who succeeds against the odds can boost motivation for personal goals [53].

#### Designing the narrative

The narrative explicitly followed the sequence of the behaviour change strategy from motivation through action to maintenance of reduced drinking. It described the journey of a central character, Dave, as he decided that his drinking was a problem and moved from regular binge drinking to moderated consumption. The narrative also included Dave’s wife Christine and a few of his friends (Table 1). The number of named characters, and the background information on each, was limited to ensure that the study participants could follow their individual stories. The narrative was written out in full before considering how it could be rendered into text messages.

**Table 1:** Characters in the narrative

Characters in the narrative	
Main character: Dave is a family man who is married to Christine. He initially believes he is a ‘mature’ drinker. He subsequently realises that he is a regular binge drinker and becomes aware of the potential risks from his drinking. He models behaviour change techniques that are likely to work but also experiences lapses along the way. In the end, he achieves his goal to cut down on his drinking and is satisfied with the outcome of the changes he has made.	
Other characters (Dave’s friends): Stevie is the unmarried ‘antagonist’ character. He has few responsibilities; he is unemployed and lives with his mother. Stevie often encourages everyone around him to drink. Dougie has had serious alcohol-related problems in the past. He lives with a long-suffering partner (Sadie) but has a troubled relationship. He also tries to change his drinking but gets it wrong more often than Dave. Alec was previously a heavy drinker but is now a mature, sensible drinker. He is respected by the others and is a role model for Dave.	

The characters were designed to make them credible to the participants [54] so they could form personal connections with them [55]. Dave was presented as someone who believed he was a mature drinker (a family man with responsibilities) but who was still binge drinking frequently. He was designed to be likeable and as someone who would succeed in changing his behaviour. However, so that the participants would empathise with his experiences, he also faced disappointment and failure before finally achieving his goals. Dave’s fallibility was intended to encourage men to identify with him and his resilience to inspire them. He modelled the process of reflection on his drinking to encourage participants to review their own behaviour, motivations and circumstances. The steps in the behaviour change strategy that Dave demonstrated are shown in Table 2. Using Dave to model these steps allowed the intervention to focus on specific rather than general behaviours and to set them in a social context familiar to participants. This process may engender self-efficacy through vicarious experience, particularly if the men identify with the characters in the narrative.

**Table 2:** Steps to behaviour change modelled in the narrative

Modelled by Dave	
Self-monitoring of drinking	
Risk perception	
Changes in outcome expectancies for heavy/binge drinking	
Increasing intention to reduce drinking	
Subjective norms	
Goal setting	
Action planning	
Increasing action self-efficacy	
Benefits of success at sticking to the plan	
Relapse	
Coping planning	
Coping self-efficacy	
Satisfaction with changed drinking pattern	
Maintenance from benefits of achieving goals	

Dave’s friends, like the protagonist, were also people with whom the participants could identify. They differed in drinking patterns (e.g. previous heavy drinker, regular heavy binge drinker) and in their demographic characteristics (i.e. employed/unemployed, single/in a relationship/family man). An important role of the characters was to make the harms of alcohol more relevant and concrete to the participants. These characters were more likely to fail in achieving their goals, a feature intended to promote empathy and elicit sympathy from participants. One character, Alec, the previous heavy drinker, was presented as an admirable role model.

Narratives frequently evoke emotional responses, and these can have strong effects over and above more rational cognitive approaches [55]. Thus emotive topics were used to increase motivation to change, e.g. one of the character’s partner and child leave home because of problems caused by alcohol. A second character, an irresponsible drinker, finds a partner at the end of the narrative because of the successful efforts he has made to reduce his drinking.

#### Review of content

To assess the completeness of the provisional intervention, the sections of the narrative were reviewed to ensure that the key components of the behaviour change strategy were contained in the narrative. This also identified whether the behaviour change techniques were appropriate for the proposed mechanism of change.

### Stage 3: Drafting the text messages

The text messages were constructed so that the main character, Dave, appeared to be a recipient of the intervention. Thus, he commented on the text messages, answered questions and modelled behaviours that were expected from the behaviour change strategy (Table 2). This avoided didactic delivery of the intervention, which preliminary work in the feasibility study had found to be unwelcome. To simplify the narrative, Dave was the only character who sent messages, although he discussed at length what was happening in the lives of the other characters. Messages containing a narrative were identified either by Dave introducing himself or signing off at the end of the message.

The complete intervention was rendered in a series of 112 text messages, each with one or more of the following purposes:

- Delivering the narrative
- Increasing the salience of the harms of heavy drinking and the benefits of moderated drinking
- Modelling steps in the behaviour change process
- Giving information or facts (to augment the behaviour change strategy portrayed in the narrative)
- Asking questions (to encourage reflection and increase the impact of intervention components)
- Increasing the impact of components of the intervention by using anonymised quotes from the feasibility study participants
- Adding humour (to increase engagement)

#### Design decisions

Several studies have identified features which need to be addressed when designing text message interventions [56–58]. These include the duration of the intervention, the frequency of sending texts, tailoring of messages to individuals, the informality of the language used and the extent of interactivity. In addition, the prior feasibility study showed that the use of linked text messages and direct quotes from men in the target group were useful techniques [43, 44].

##### Specify dates and times to send messages

The messages were tailored to the day of the week. Thus, the intervention was designed so that the first text message would be sent on the Monday evening following randomisation. The feasibility study revealed that a common pattern is heavy drinking at the weekend followed by sobriety during the week. Text messages sent on Friday and Saturday were therefore delivered in the afternoon or early evening before the men went out drinking. Messages sent on Sunday were generally delivered later in the evening to give the participants a little more time to recover from a hangover. Mid-week text messages were sent at variable times, often after the working day.

The previous research offers differing views on message frequency. One systematic review suggests that retention is higher if the number of messages is varied over time [59], while another reported that interventions when message frequency decreased were more effective than those with constant frequencies [37]. All of the messages in this intervention were unique, although some topics, e.g. self-efficacy and maintenance of a new behaviour were revisited at different stages of the intervention. The intervention was designed so that participants would receive at least one message every day for the first 5 weeks. The maximum number of messages sent in a day was four. From week 6 onwards, occasional days were missed.

##### Use of linked messages

The text messages were often sent in pairs or groups of three or four. This device has several purposes. Linked messages enabled more complex messages to be sent as some of the reflective activities and behaviour change strategies could not be explained in a single message. They were also used to extend the time the participants had to think about a topic. The first message was often used to seed an idea, while the follow-up text messages encouraged reflection on the topic. Combinations of messages could add suspense and build a storyline. Responses from characters in the narrative were used to illustrate how the messages could be interpreted (e.g. for goal setting or action planning) or to give examples of reasons for changing behaviour. Paired messages could also pose a question, with the answer provided later in the day. The time delay between linked messages varied from 3 min to 4 h.

##### Making the intervention acceptable

Communication theory [60, 61] was used to enhance the acceptability of the intervention. The name of the university was used to give credibility to the study and the intervention. It was mentioned on all written material given to the men during recruitment. To establish a relationship, participants were sent a welcoming text message which included their first name. Text messages did not include messaging slang as it could be construed as unprofessional coming from a credible source, i.e. a university. Communication theory suggests that interesting and unexpected statements can be used to maintain interest. Thus, humour was used throughout the intervention period.

#### Techniques to increase engagement with the texts

##### Interactive items for key components of the intervention

The use of interactive text messages was central to the intervention. Mobile phone etiquette requires reciprocation, so that messages which ask questions are likely to be answered [62]. The target group are frequent mobile phone users and therefore likely to engage in text message conversations. This was capitalised on by asking questions on the key components of the behaviour change strategy. For example, participants were asked: ‘If you made a goal to cut down a bit on your drinking, what would it be? Text me your answer’ or ‘What would you do if you got into a situation where you were expected to drink far more than you intended? Text me your answer’. The responses to these questions provided an indication of engagement with the intervention in real time. The feasibility study showed that participants engaged with the cognitive antecedents to reducing drinking and with important steps on the causal chain to behaviour change [44].

##### Quotes from the feasibility study

The feasibility study produced a wealth of data ‘in the participants’ own words’ both from focus groups and text message responses from those who received the intervention [43]. Several texts from that study were presented as anonymised quotes from individuals describing their personal experiences. For example, to increase perceived risks of heavy drinking, one message said “John from Dundee says ‘I’ve woke up in the cells a few times because of drink. if i was sober it would never have happened’”. This technique was used to illustrate harms from alcohol misuse, to model new behaviours and to report achievements and benefits from changing behaviour. The quotes delivered information in the language used by the target group and were intended to encourage participants to share their own experiences. To reinforce their authenticity, the quotes were not corrected for spelling or grammar.

### Stage 4: Revision of the text messages

#### Ensure coherence of the narrative and the behaviour change strategy

When the intervention had been rendered into text messages, it was reviewed to ensure that it was complete and coherent and that all of the components of the intervention had been included (i.e. the key components of HAPA, the behaviour change techniques, BCTs). Initially, the broad structure of the intervention was clarified by grouping text messages by their intended function, identifying the logical progression through the intervention (Additional file 2: Table S1). Then, individual text messages were mapped to components of the HAPA model and to specific BCTs (Additional file 2: Tables S2 and S3). Finally, the narrative text messages were mapped to ensure the story was coherent (Additional file 2: Table S4).

The messages were also read by colleagues who knew the storyline of the narrative and the behaviour change techniques and processes that should be incorporated into the intervention. They were asked to establish whether each component of the behaviour change strategy was addressed in sufficient detail. They also checked whether the reader could follow the narrative when it was presented as a series of text messages. This process was then repeated with colleagues unfamiliar with the narrative and the intervention. Ambiguous statements were modified to ensure that the unedited direct quotes from the feasibility study were easily understood.

#### Piloting and final revisions

Guidance on text message intervention development suggests that rigorous pretesting should be done with the target group [63], to ensure the messages are relevant and have the intended impact [64]. The final piloting used 24 volunteers (8 members of the target group, 13 post-graduate students and 3 members of university staff unconnected with the study). They were given background information on the study and were told that the characters in the narrative were fictional. These volunteers received a copy of the text messages on paper and were asked to provide written comments, both on the overall approach and on individual text messages. All eight of the target group members engaged with the characters and the narrative as if they were real and responded directly to the text messages rather than commenting on their appropriateness of their content. The post-graduate students and staff approached the task as an academic exercise and commented on the readability and potential impact of the texts. However, one student changed roles partway through and began responding to the texts as if he were a participant.

The volunteers’ comments gave reassurance that most texts were clear and readily understood, although a few needed rewording. No one identified components of the intervention which were missing, inappropriate or inadequately addressed. The comments showed that the use of characters made the intervention appear more realistic and less daunting. The volunteers also found the overall approach supportive. Finally, they suggested how the narrative be amended. Several students expressed concern about the fate of one character, Stevie, so the narrative was amended to give him a happy ending. A second round of piloting involved new volunteers (three members of the research team) who received the text messages on their mobile phones. This was primarily used to test the delivery system but also helped confirm that the frequency and timing of the messages were acceptable. The final version of the text messages is supplied as Additional file 1 (Intervention text messages).

## Discussion

A systematic approach based on theory, evidence and formative research was used to design a tailored text message intervention to reduce binge drinking among socially disadvantaged men. Following the MRC framework guidance [18], the intervention was developed from a review of the target group, the health behaviour being studied and selection of the most appropriate psychological model and behaviour change techniques. The findings were then used to create a logic model exploring how the intervention strategy could lead to the desired short- and long-term outcomes. This led to a decision on the delivery method and text messages and to formative research to inform intervention development. A revised intervention incorporating a narrative was developed and fine-tuned in further pilot testing.

The decision on the delivery method, text messages, was made because of concerns about the recruitment and engagement of disadvantaged men in the study. Socially disadvantaged people are seen as a hard-to-reach group and reluctant to take part in research [65, 66]. However, they are the most frequent users of mobile phones and are more likely to use text messaging and send and receive a higher number of text messages than people with higher education and income [67, 68]. A text message intervention, which did not require any face-to-face contact with researchers or clinicians, was thought likely to engage the target group. Further, a feature of text messaging etiquette is that texts are likely to prompt a response [62]. Thus, questions were regularly asked on the key components of the HAPA model to foster engagement. Text message questions have been used in previous trials to promote interactivity [56, 57], but this is the first study to report using this device as part of the intervention.

The novel feature of the design was the use of narrative in a text message intervention. This was suggested by one of the authors (BW) to provide a structure around which text messages could be woven, increasing coherence and sustaining the interest of participants. However, a narrative-based intervention also brings additional benefits of increased effects on knowledge, attitudes and intentions compared to the same information in a non-narrative format [47]. Narrative is particularly useful for changing perceived social norms and behavioural intentions [48, 49]. The use of a narrative also provided an opportunity to present the intervention in a non-patronising way. A major advantage of the narrative was that the lead character, Dave, could model key behaviours such as action planning, relapse recovery and coping planning. Including questions in the text messages reinforced this by asking participants to explain how they would perform similar actions [69].

This study has shown that a narrative-based intervention, which covers the components of a psychological model (HAPA), can be fully addressed in a text message intervention. Even complex psychological constructs can be conveyed through a few linked text messages through a careful process of design, piloting and testing. A potential challenge arose from the process in which the behaviour change strategy had to be translated into a narrative which then had to be rendered in text messages. As elements of the intervention could be omitted at either step, repeated checking was carried out to ensure fidelity with the initial design.

### Limitations of the study design

Identifying men who are socially disadvantaged may be challenging. Using an area-based measure of social disadvantage may include individuals living in affluent areas which are located within postcode areas that are classified as being socially disadvantaged. Tailoring interventions to a large potentially heterogeneous group is challenging. This was addressed through extensive piloting during development.

Delivering the intervention over a 12-week period could be seen as a weakness, by diluting the intensity of intervention delivery. However, the extended time allowed participants to reflect on the content over a longer period. This approach enabled the key components of the intervention to be revisited so that commitment to change could be reinforced.

The intervention was designed to encourage the participants to respond to text messages. However, this intervention cannot be truly interactive, i.e. participants will not receive replies to their responses, except in exceptional circumstances, for example if they report distress. Failure to receive replies may discourage participants to respond as the intervention progresses.

A constraint in writing the text messages was the permitted length of a text (160 characters). Thus, the storyline had to be fairly simple and straightforward to be delivered in a few words. A feature of a narrative is that it should provide sufficient context and information for participants to have an understanding of the scenario being depicted but needs to be vague in ways that encourage participants to fill in the detail using their imagination and based on their own life experience [54]. Thus, the scant information presented within the text messages may encourage participants to embellish the storyline to make it fit their personal social circumstances.

Delivering interventions by mobile phone may have limitations. Some participants may not receive messages if they have a weak phone signal or if their phones are continually switched off. Having insufficient credit may deter participants from engaging with the intervention and some may delete messages without reading them. This study was designed to prompt responses so that we could monitor whether participants were opening and reading the text messages.

Adding humour was used to maintain interest in the text messages. Although our feasibility study and piloting of the intervention showed that the jokes were well received, it is possible that in a large study, some individuals might dislike the jokes. In general, humour should be used with care.

## Conclusion

This structured, systematic approach has led to the design of a text message intervention with a strong theoretical and empirical basis. The process was highly iterative to enable a theory of behaviour change and a set of behaviour change techniques to be embedded in a coherent narrative. These were successfully rendered in a series of short text messages. The use of a narrative helped make the intervention realistic and allowed key behavioural activities to be modelled by characters. Pilot testing revealed strong support for the intervention. This approach provides a framework for the design of behaviour change interventions which could be used for interventions to tackle other health behaviours.

## Additional files

Additional file 1: Intervention messages. (DOCX 35 kb)

Additional file 2: Mapping the text message intervention to alcohol brief interventions (ABIs), the Health Action Process Approach (HAPA), Behaviour Change Techniques (BCTs) and the narrative. **Table S1.** Intended purpose of the groups of text messages. **Table S2.** Mapping text messages onto the components of Alcohol Brief Interventions. **Table S3.** Mapping of text messages onto the components of HAPA. **Table S4.** Behaviour change techniques to reduce excessive alcohol consumption. **Table S5.** Mapping of the text messages onto the narrative. (DOCX 30 kb)

## Abbreviations

ABI: Alcohol brief intervention
BCT: Behaviour change technique
HAPA: Health Action Process Approach
MI: Motivational interviewing
