# Supplementary material for: Design and development of a complex narrative intervention delivered by text messages to reduce binge drinking among socially disadvantaged men
Source: Pilot Feasibility Stud. 2018 Jun 6;4:105. doi: 10.1186/s40814-018-0298-0 (PMC5989343; doi:10.1186/s40814-018-0298-0)
Supplement: Supplementary file 1 — Intervention messages. (DOCX 35 kb) [file 40814_2018_298_MOESM1_ESM.docx]

**Intervention messages**

| **Week** | **Day Number** | **Day** | **Text Number** | **Time** | **Message** |
| --- | --- | --- | --- | --- | --- |
| 1 | 1 | Monday | 1 | 18.00 | Welcome <<name>>. Thank you for helping with this important study on alcohol. Over the next three months we’ll send you some text messages |
| 1 | 1 | Monday | 2 | 18.05 | Some texts give info on alcohol. Some ask about you. You’ll also hear from Dave and other men. These are characters based on our research with men in Scotland. |
| 1 | 2 | Tuesday | 3 | 13.00 | Hi, I’m Dave. I’m doing this with the Uni too. Might be good for a laugh. I’ll also send you some texts. You’ll maybe hear from my mates Stevie and Dougie too. |
| 1 | 3 | Wednesday | 4 | 19.45 | We all drink for different reasons. What’s the main reason you drink? (a) it’s a habit (b) to feel better (c) to have fun (d) to cope. Text me your answer. |
| 1 | 4 | Thursday | 5 | 18.00 | It’s Dave here. Just seen that message about why we drink. I drink to enjoy myself. Gone are the days when I got wasted as often as possible and for no reason. |
| 1 | 4 | Thursday | 6 | 19.00 | Thinking about it, my mate Stevie is always tanking it. For him it has to be a habit. Cans in the house all day some days. Can’t just be for fun at his age. D. |
| 1 | 5 | Friday | 7 | 13.30 | Warning: Drinking alcohol may cause you to roll over and see something really scary (whose species or name you can’t remember). |
| 1 | 5 | Friday | 8 | 13.35 | Nice one. Done that, less said the better. Cheers Dave |
| 1 | 6 | Saturday | 9 | 15.00 | How often do you end up drinking far too much at a friend’s house or in the pub when you really meant to take it easy? You’re not alone. It happens a lot. |
| 1 | 7 | Sunday | 10 | 19.15 | Did you know heavy drinking messes with your hormones and can give you man boobs or male breasts? Stay moob free. |
| 1 | 7 | Sunday | 11 | 19.35 | I need to watch out. Have to admit, I’m putting on a bit. Didn’t know about hormones. I thought it was just women who were ruled by hormones. Dave |
| 2 | 8 | Monday | 12 | 16.00 | Do you try to keep track of what you drink on a night out? It can be hard. What’s your usual amount? Text me your answer. |
| 2 | 9 | Tuesday | 13 | 19.35 | I drink at the weekend and after Thursday footie. That’s most of the working week alcohol free. A lot less than back in the day before kids and regular work. D. |
| 2 | 9 | Tuesday | 14 | 20.05 | Talking to my mate Stevie about these texts. Says he couldn’t keep track if he tried. Maybe he should. He’d get a surprise. D. |
| 2 | 10 | Wednesday | 15 | 16.30 | Just so you know, binge drinking is more than 8 units in a session. A measure of spirits is 1 unit and a pint of beer or lager is well over 2 units. |
| 2 | 10 | Wednesday | 16 | 16.34 | Be thankful you’re not a woman – binge drinking is 6 units for them. |
| 2 | 11 | Thursday | 17 | 18.15 | Dave here. Did you see that? Binge drinking is 8 units in one go – can’t get pissed on that. I thought binging meant putting away litres of cider or vodka. |
| 2 | 11 | Thursday | 18 | 19.30 | I thought I was fine. Maybe I need to add up what I do get through in a weekend. D. |
| 2 | 12 | Friday | 19 | 16.15 | Try to keep track of what you drink this weekend. Write down the total or note it on your phone. It can be hard to remember if you’ve had a few. |
| 2 | 13 | Saturday | 20 | 15.00 | Things impossible to say when drunk. (a) Thanks, but I don’t want to sleep with you (b) How’s it going officer? (c) No kebab for me thanks. |
| 2 | 14 | Sunday | 21 | 20.30 | Did you manage to count how much you drank over the weekend? Text me your answer. |
| 3 | 15 | Monday | 22 | 18.30 | Done the maths. Thurs: 4 pints (9 units). Fri: 6 pints, (14 units). Sat: 3 or 4 pints and a few drams (say 12 units). Sun: maybe 4 cans (9 units). Dave. |
| 3 | 15 | Monday | 23 | 20.00 | That’s binge drinking 4 nights out of 7. Never looked at it that way before. That’s more than 40 units in a week. Now that is a shock for me. D. |
| 3 | 16 | Tuesday | 24 | 14.00 | Can you think of any reasons why it might be a good idea for you to cut down a bit on your drinking? Please text me your answer. |
| 3 | 16 | Tuesday | 25 | 18.15 | Good reasons for me to cut down: see the kids more; keep the wife happy; more money; lose a bit of weight. Dave |
| 3 | 16 | Tuesday | 26 | 19.05 | Told Stevie a good reason for him would be holding on to a girlfriend for more than a week. And he agreed! Dave |
| 3 | 16 | Tuesday | 27 | 19.30 | Stevie says NOT getting a beer gut like me is a more important for keeping a girl. Can’t argue with that. So, a win win situation if he cuts back a bit. D |
| 3 | 17 | Wednesday | 28 | 15.30 | Question: What’s a man’s idea of a balanced diet? |
| 3 | 17 | Wednesday | 29 | 19.30 | Answer: Having a pint in each hand |
| 3 | 18 | Thursday | 30 | 16.15 | Have you or your mates had any problems caused by alcohol? Please let me know. We’ve all been there. |
| 3 | 18 | Thursday | 31 | 18.30 | I done things I regret. Lost licence at 19 (and my job). First wife left me after 9 months (fortunately no kids then). Ended up in many dodgy places. Dave. |
| 3 | 18 | Thursday | 32 | 18.55 | John from Dundee says “I’ve woke up in the cells a few times because of drink. If I was sober it would never have happened”. |
| 3 | 18 | Thursday | 33 | 18.58 | Mark from Edinburgh says “Sometimes I’ve not had enough money left to pay the bills”. |
| 3 | 19 | Friday | 34 | 13.00 | Just spoke to Dougie. His partner wants another kid, but it’s not happening. Seems like alcohol affects that kind of performance too. Dave |
| 3 | 20 | Saturday | 35 | 11.00 | Pete from Wick says: It was the death of my father. Out drinking every day then home to drink at night and then he died. Don’t want to put myself through that. |
| 3 | 21 | Sunday | 36 | 20.00 | In the past week have you thought about cutting back a bit on your drinking? Text me (a) yes (b) no (c) maybe |
| 4 | 22 | Monday | 37 | 18.30 | Dave here: Dougie’s partner has taken the kid and gone to her mum’s. Says she’s fed up with his drinking and having no money to get the kid any treats. |
| 4 | 22 | Monday | 38 | 18.33 | My wife wasn’t surprised and even said “well done Sadie”. That’s a bit of a shocker. Dougie’s not that different from me these days. I need to watch my step. D. |
| 4 | 23 | Tuesday | 39 | 16.00 | Thinking about cutting back? Still undecided? Don’t worry. It takes time to weigh things up. |
| 4 | 24 | Wednesday | 40 | 19.30 | Hey it’s Dave: Been thinking about cutting back. My friend Alec always asks–“Done anything with the kids this weekend?” I’m sure it’s a hint. |
| 4 | 24 | Wednesday | 41 | 19.35 | I call Alec the Major. Ex-army, hard as nails. Lost his kids for a while. Turned things round. Dotes on grand-kids now. Tries to keep us right. D. |
| 4 | 25 | Thursday | 42 | 19.00 | Can you think of someone who’d be happy if you made a change? What would you hear them say? Please text me your answer. |
| 4 | 26 | Friday | 43 | 18.30 | I know for a fact Christine (the wife) would be all for me cutting down, and so would my dad. Dave |
| 4 | 26 | Friday | 44 | 20.30 | Stevie says his mum would say “it’s great not having to deal with a drunken ass at 2am”. |
| 4 | 27 | Saturday | 45 | 17.00 | Knowing your limits is a good way to stay in control and make sure you don’t overdo it. |
| 4 | 28 | Sunday | 46 | 18.30 | Many people want to cut back a bit. Can you tell me what would be good about that for you? |
| 4 | 28 | Sunday | 47 | 19.30 | Dave again: More money, more time, maybe lose a bit of weight. |
| 5 | 29 | Monday | 48 | 18.00 | How much would you save each month if you drank half as much? |
| 5 | 29 | Monday | 49 | 18.03 | Count up your savings and text me the sum. |
| 5 | 30 | Tuesday | 50 | 18.15 | I’m getting good at sums with all these questions. It’s like being back at school. I’d have saved £100. I won’t be telling Christine that. Dave |
| 5 | 30 | Tuesday | 51 | 18.45 | Stevie says he’d save much more than that. Reckons he could afford a car. Now that would impress the girls. He’d have to pass his test first though. D |
| 5 | 30 | Tuesday | 52 | 19.00 | By saving your cash you could treat yourself to something special too. Picture what you would like and text me back your answer. |
| 5 | 31 | Wednesday | 53 | 15.00 | We’ve all got our routines and changing them can be tricky. Setting yourself a goal helps and gives you something to focus on. |
| 5 | 31 | Wednesday | 54 | 15.10 | The best goals say exactly what you want to achieve. Straight to the point. Make it a challenge but not impossible. |
| 5 | 31 | Wednesday | 55 | 19.15 | OK, let’s do it. My goal is to lose 2 of the heavy sessions this week. I’m sure I can take it down a notch or two. Time to man up and cut down a bit. Dave |
| 5 | 32 | Thursday |  |  |  |
| 5 | 33 | Friday | 56 | 16.30 | If you made a goal to cut down a bit on your drinking, what would it be? Text me your answer. |
| 5 | 33 | Friday | 57 | 19.15 | Mark from Methil says “By the end of the month, I’m going to cut out the cans in front of the telly on a Sunday night.” |
| 5 | 34 | Saturday | 58 | 12.00 | Rob from Govan says “My goal would be to get hame withoot forgettin where ive been or how I got there”. |
| 5 | 35 | Sunday | 59 | 20.30 | How confident are you that you could cut back a bit? (a) absolutely certain (b) pretty sure (c) maybe (d) no chance. Text me back please. |
| 6 | 36 | Monday | 60 | 16.00 | A good way to keep on track is to plan ahead on how you will achieve your goal. |
| 6 | 36 | Monday | 61 | 18.30 | When you make a plan it always works better if you make sure you say: WHEN; WHERE; and HOW you will do it. |
| 6 | 36 | Monday | 62 | 18. 55 | I like a challenge so I’ve made a plan: WHEN: Friday. WHERE: Home for tea before the pub. HOW: Less time to have a skinful. Dave |
| 6 | 37 | Tuesday |  |  |  |
| 6 | 38 | Wednesday | 63 | 18.35 | I’ve made another plan: Saturday night. WHERE: the club. HOW: Drink at Alec’s pace not Stevie’s. And I’ll tell Stevie and Doug what I’m doin. D. |
| 6 | 38 | Wednesday | 64 | 19.00 | If you made a plan, what would it be? Text me your answer. |
| 6 | 39 | Thursday | 65 | 15.15 | Mark from Methil says “A plan for this weekend. I’m going to have fewer cans in the fridge at home”. |
| 6 | 39 | Thursday | 66 | 17.30 | So for Mark the plan is: WHEN: This weekend. WHERE: At home. HOW: Fewer cans in the house to drink. |
| 6 | 40 | Friday | 67 | 18.00 | Let friends & family know if you’re trying to cut down. Tell them about this study and they may give you their support. |
| 6 | 41 | Saturday |  |  |  |
| 6 | 42 | Sunday | 68 | 19.30 | Dave here. Stuck to the plan all weekend. Paid off – definitely a few less on both nights. Felt better. Took the kids swimming. Nice dinner with Christine. |
| 7 | 43 | Monday | 69 | 14.00 | Don’t worry if you feel you’re not doing enough. Even a little less is a good thing. Just keep trying. It will pay off. |
| 7 | 44 | Tuesday | 70 | 13.00 | Plan went out the window last night. Stevie’s birthday. Got absolutely steamin. Good fun, but got more than a few headaches today. Dave |
| 7 | 44 | Tuesday | 71 | 18.00 | Even if things come unstuck and you end up pissed, don’t make a big deal of it. It happens. Don’t dwell on it. See if you can learn from it. |
| 7 | 44 | Tuesday | 72 | 18.30 | Thinking about it now I should have seen it coming. Stevie’s birthday, so he was calling the shots. No pun intended! Dave. |
| 7 | 45 | Wednesday | 73 | 14.30 | What would you do if you got into a situation where you were expected to drink far more than you intended? Text me your answer. |
| 7 | 45 | Wednesday | 74 | 18.35 | Seems obvious now. In my situation it would have helped to have a wee plan. That’s worked before. After a skinful there’s no way I can think on my feet. D. |
| 7 | 46 | Thursday | 75 | 13.00 | Don’t let a slip get you down. These things happen. Best thing to do is get another plan and carry on. |
| 7 | 47 | Friday | 76 | 19.30 | Time to get back on track. Just because I had a blow out with Stevie once doesn’t mean I stop trying. I’m no quitter. It worked pretty good last time. Dave |
| 7 | 48 | Saturday | 77 | 14.30 | Making plans to deal with tricky drinking situations can help you stay in control. |
| 7 | 49 | Sunday | 78 | 19.45 | If we win a match (happens occasionally), we celebrate by buying rounds. Next time I’ll stick to half pints. I’ll say I want to lose weight – that’s true. Dave |
| 7 | 49 | Sunday | 79 | 19.50 | And I might have Alec and his missus round on a Saturday instead of going to the pub. I’d drink less and would feel better for it on Sunday. D |
| 8 | 50 | Monday | 80 | 19.00 | Would you feel comfortable about refusing a drink when you’re out with your mates and drinking in rounds? Text me back please. |
| 8 | 50 | Monday | 81 | 20.15 | Joe from Perth says: When I was younger, you couldn’t say no, I’ll have a coke. You’d probably get abuse for it. Now me and my mates are older it’s acceptable. |
| 8 | 50 | Monday | 82 | 20.25 | Stuart from Brechin says; I’ve done it if I’ve felt I’ve had too much or I’ll just miss one when I’ve finished my drink. |
| 8 | 51 | Tuesday | 83 | 17.00 | If a plan worked for you before, it will work again. Remember WHEN; WHERE and HOW, helps you to make a plan that can work. |
| 8 | 52 | Wednesday | 84 | 18.30 | If you had an unplanned binge, how confident are you that you could get back on track next time? (a) absolutely certain (b) pretty sure (c) maybe (d) no chance |
| 8 | 53 | Thursday | 85 | 19.15 | Have you thought about doing something different with your time? What could you do to avoid a drinking session? Text me your answer. |
| 8 | 53 | Thursday | 86 | 19.30 | I go swimming every week. Started goin with the kids. Now I practice one night on my own so that I can still beat them. Dave |
| 8 | 54 | Friday | 87 | 18.45 | Drinking too much can spoil a good night and make you regret things you did. Pacing yourself makes for a better night. |
| 8 | 55 | Saturday | 88 | 14.30 | When you try to cut down a wee bit, it’s just a case of keeping trying. You’ll find it works most of the time. |
| 8 | 56 | Sunday | 89 | 18.30 | Stevie’s learnin to cook. He’s been watching TV chefs and cooking for his girl. Cooking and eating instead of drinking. For Stevie that is impressive. D |
| 9 | 57 | Monday | 90 | 19.15 | Getting a result from changing things makes the effort involved worthwhile. What would make it worthwhile if you cut back a bit? Text me your answer. |
| 9 | 58 | Tuesday | 91 | 13.00 | Losing weight definitely made it worthwhile for me. Didn’t change what I ate, so cutting the number of pints must have done it. Dave |
| 9 | 58 | Tuesday | 92 | 20.15 | Dave again: Kids tell me I’ve lost my man boobs – down to a C cup now! That’s certainly a result. Christine is impressed. |
| 9 | 59 | Wednesday |  |  |  |
| 9 | 60 | Thursday |  |  |  |
| 9 | 61 | Friday | 93 | 19.00 | Think of the reason you wanted to cut down and remember it when you want to have that extra pint or three. |
| 9 | 61 | Friday | 94 | 19.10 | Neil from Kilmarnock says “I could afford to go to the pictures twice this week. That pleased my girlfriend”. |
| 9 | 62 | Saturday | 95 | 16.00 | I’ve learned just to keep at it. Like staying in control when things are getting silly in the pub. Dave |
| 9 | 62 | Saturday | 96 | 16.10 | Always glad when I do. The thought of feeling crap afterwards is enough these days. Getting wasted was crap – even though I didn’t know it at the time. D |
| 9 | 63 | Sunday |  |  |  |
| 10 | 64 | Monday | 97 | 15.15 | In the past 3 months have you (a) thought about changing your drinking (b) set a goal to change it (c) made a plan of how to do it (d) never thought about it? |
| 10 | 65 | Tuesday | 98 | 19.20 | If you have cut down on the drinking a bit, have you noticed any differences to you or your family? Let me know please. |
| 10 | 66 | Wednesday | 99 | 13.00 | We’ve all noticed a difference on the football pitch. It is definitely easier to run about. Dave |
| 10 | 66 | Wednesday | 100 | 19.30 | I’m impressed by Stevie. He’s taken this onboard too. He’s looking sharp for the first time in years and his girlfriend is lovin the new image. Dave |
| 10 | 67 | Thursday |  |  |  |
| 10 | 68 | Friday | 101 | 16.00 | Think about times when you are tempted to drink far too much. What could you do to stop it happening? Text me your answer. |
| 10 | 68 | Friday | 102 | 19.30 | Dougie says: Thinking about how my drinking will hurt people around me would work. |
| 10 | 68 | Friday | 103 | 19.45 | Buying something nice for yourself for making the effort to change also works. |
| 10 | 69 | Saturday |  |  |  |
| 10 | 70 | Sunday |  |  |  |
| 11 | 71 | Monday | 104 | 11.30 | Jim from Falkirk says: “I’m stayin out of trouble and not becoming the person I can after a few too many”. |
| 11 | 72 | Tuesday |  |  |  |
| 11 | 73 | Wednesday | 105 | 19.30 | Dave again: These days I don’t mind telling the guys where to go when they want to go on a bender. It’s good knowing when to stop. |
| 11 | 74 | Thursday |  |  |  |
| 11 | 75 | Friday | 106 | 18.30 | Reward yourself when you stick to your goal. Use the money you save. Savings mount up. In a few months could it be; new jeans; a laptop; a telly; a holiday? |
| 11 | 76 | Saturday | 107 | 12.00 | Dave here: Good stuff. I’m savin for a 50” telly. Not Christine’s first choice, but the kids will love it. |
| 11 | 77 | Sunday |  |  |  |
| 12 | 78 | Monday |  |  |  |
| 12 | 79 | Tuesday |  |  |  |
| 12 | 80 | Wednesday | 108 | 19.30 | That’s been a few weeks now without any major slip–ups. I’ll never be perfect, but you just need to keep at it. Dave |
| 12 | 80 | Wednesday | 109 | 19.35 | Now when I drink it’s because I feel good about myself. That way, I relax at the end of the week, have a drink, knowing that everything is as it should be. D |
| 12 | 81 | Thursday |  |  |  |
| 12 | 82 | Friday | 110 | 17.00 | Congrats <<name>>, you’ve completed the study. A million thanks for taking part. We’ll be in touch again in 3 months to see how you’re getting on. |
| 12 | 83 | Saturday | 111 | 12.00 | Dave signing off. Hope you’ve enjoyed this as much as me. Things are looking better for me. For you too I hope. |
| 12 | 84 | Sunday | 112 | 19.00 | Please keep in touch. When we call you back, you’re due another £10 voucher. If you change your phone number, please send us your new one. |
|  |  |  |  |  |  |
|  |  |  |  |  |  |
| 24 | 164 | Wednesday | 113 | 18.00 | Hi <<name>>. That’s nearly three months since we were in touch. Hope you’re doing well. We’ll phone soon to hear how you’ve been getting on. |
| 24 | 164 | Wednesday | 114 | 18.10 | Forgot to say, let us know if there’s a good time to call. Remember, you’ll get another £10 voucher. |
| 24 | 165 | Thursday | 115 | 19.30 | Good to hear from you. Missed the texts comin in. I’ll look out for the call. Cheers mate. Dave |
| 24 | 165 | Thursday | 116 | 19.35 | Should have said. Got my telly last week. Guess who watches it most! Correct. The missus! Soaps every night. What a waste. Dave. Oh, and Stevie just got engaged |
|  |  |  |  |  |  |
| 64 | 446 |  | 117 | 19.00 | Hi <<name>>. Do you remember taking part in our study last year? I hope you are getting on well. We’ll phone again soon to hear how you’ve been getting on. |
| 64 | 446 |  | 118 | 19.03 | Please let us know if you’ve changed your address. We need it to send out the last £10 voucher when we’ve had a chat on the phone. |
