# Supplementary material for: Design and development of a complex narrative intervention delivered by text messages to reduce binge drinking among socially disadvantaged men
Source: Pilot Feasibility Stud. 2018 Jun 6;4:105. doi: 10.1186/s40814-018-0298-0 (PMC5989343; doi:10.1186/s40814-018-0298-0)
Supplement: Supplementary file 2 — Mapping the text message intervention to alcohol brief interventions (ABIs), the Health Action Process Approach (HAPA), Behaviour Change Techniques (BCTs) and the narrative. Table S1. Intended purpose of the groups of text messages. Table S2. Mapping text messages onto the components of Alcohol Brief Interventions. Table S3. Mapping of text messages onto the components of HAPA. Table S4. Behaviour change techniques to reduce excessive alcohol consumption. Table S5. Mapping of the text messages onto the narrative. (DOCX 30 kb) [file 40814_2018_298_MOESM2_ESM.docx]

**Mapping the text message intervention to Alcohol Brief Interventions (ABIs), the Health Action Process Approach (HAPA), Behaviour Change Techniques (BCTs) and the narrative**

This document maps individual text messages to their functions. The intervention was structured around a behaviour change theory (the Health Action Process Approach, HAPA [1]) and incorporated BCTs identified as appropriate for alcohol interventions [2] and elements from Alcohol Brief Interventions[3]. The intervention was embedded in a narrative, in which characters model many of the activities involved in the process of behaviour change.

The mapping process is a complex one. The conventional length of a text message, 160 characters, is insufficient to convey a construct from HAPA, a BCT or an element from Alcohol Brief Interventions. Instead complex elements were developed over several texts and frequently reinforced later in the intervention. Thus the texts were mainly written as groups of messages. Most groups involved more than one component of the intervention, for example they could include a construct from HAPA, and a BCT which is then modelled by a character from the narrative. A consequence of the reinforcement of components of the intervention is that specific HAPA constructs or BCTs are addressed by several text messages throughout the intervention.

To give an overview of the intervention, the purpose of each group text messages is stated in Table S1. To clarify the role of individual text messages we have mapped them in four ways, to:

i) the elements of Alcohol Brief Interventions structured around the elements of FRAMES (Table S2)

ii) the constructs from the HAPA model (Table S3)

iii) BCTs (Table S4)

iv) the main narrative elements (Table S5).

**References**

1. Schwarzer R. Modeling health behaviour change: How to predict and modify the adoption and maintenance of health behaviours, *Appl Psychol* 2008: 57: 1-29.

2. Michie S., Whittington C., Hamoudi Z., Zarnani F., Tober G., West R. Identification of behaviour change techniques to reduce excessive alcohol consumption, *Addiction* 2012: 107: 1431-1440.

3. Bien T. H., Miller W. R., Tonigan J. S. Brief interventions for alcohol problems: a review, *Addiction* 1993: 88: 315-335.

**Table S1: Intended purpose of the groups of text messages**

| **Topic** | **Message numbers** |
| --- | --- |
| Welcome to the study and introduce characters in the narrative | 1 - 3 |
| Drinking motives | 4 - 6 |
| Humour/outcome expectancy | 7 - 8 |
| Outcome expectancy/risk perception | 9 - 11 |
| Self-monitoring of behaviour | 12 - 14 |
| Definition of binge drinking | 15 - 17 |
| Self-monitoring of behaviour | 18 - 19 |
| Humour | 20 |
| Self-monitoring of behaviour | 21 - 23 |
| Identifying good reasons for cutting down | 24 - 27 |
| Humour | 28 - 29 |
| Identifying alcohol related harms in self and others | 30 - 35 |
| Thinking about cutting down/intention to change | 36 |
| Illustration of alcohol related harms | 37 - 38 |
| Intention to change | 39 - 41 |
| Subjective norms | 42 - 44 |
| Self-monitoring | 45 |
| Perceived benefits of cutting down/outcome expectancy/ positive reinforcement | 46 - 52 |
| Goal setting | 53 - 58 |
| Action self-efficacy | 59 |
| Action plans | 60 - 66 |
| Subjective norms/social support | 67 |
| Benefits of reducing/positive reinforcement | 68 |
| Increasing action self-efficacy | 69 |
| Illustration of relapse | 70 - 72 |
| Coping planning/recovery self-efficacy | 73 - 79 |
| Coping self-efficacy | 80 - 82 |
| Recovery self-efficacy/plan reinforcement | 83 - 84 |
| Alternatives to drinking | 85 - 86 |
| Alcohol expectancies/coping planning | 87 - 88 |
| Alternatives to drinking to avoid risky drinking situation | 89 |
| Perceived/actual benefits of cutting down | 90 - 92 |
| Self-efficacy/maintenance | 93 - 96 |
| Monitoring drinking behaviour | 97 |
| Benefits of drinking less/positive reinforcement | 98 - 100 |
| Coping planning | 101 - 105 |
| Rewards | 106 - 107 |
| Satisfaction with new behaviour | 108 - 109 |
| Thank you for taking part | 110 - 112 |

**Table S2: Mapping text messages on to components of Alcohol Brief Interventions**

**FRAMES**

| **Component of FRAMES** | **Text message number** |
| --- | --- |
| Feedback | 12,14,19,21-24,30-35,37,38,48, |
| Responsibility | 11,13,18,24-27,36,39,45,46,62,63-66, |
| Advice | * |
| Menu | * |
| Empathy | 9,39,53,67,69,71 |
| Self-efficacy | 50,53,59,60,68,69,71,73-84, 87,88,90,93,94,96,103,105 |

Note: many of the texts on self-efficacy also cover empathy

* It is difficult to allocate text messages to these categories. The text messages which come closest to these components are those that illustrate the benefits of reducing alcohol consumption (text numbers 46-52), goal setting (53-58) and action planning (60-66).

**Table S3: Mapping of text messages on to components of HAPA**

| **Key component of HAPA** | **Message number** |
| --- | --- |
| **Intention to change** |  |
| Change alcohol expectancies | 5,7-8,9-11,24-27,38,42-52, |
| Change perception of risk | 6,7,9-11,30-35,37,38, |
| Increase action self-efficacy | 36,39,40, 68,69 |
| Enlist social support/subjective norms | 40,41,42,43,44, |
| **Action planning/ Coping planning** |  |
| Increase action self-efficacy | 59,90 |
| Increase coping self-efficacy | 69,73 |
| Action control (how to cope in high risk situation) | 83,85,86 |
| Goal setting | 53-58, |
| Action plans | 60-66,77,83 |
| Enlist social support | 63,67,98 |
| **Maintenance** |  |
| Recovery self-efficacy | 71,75,76,84 |
| Increase coping self-efficacy | 80-82,87,88,93-96,103,105,108,109 |
| Action control (how to cope in high risk situation) | 72,74,77-79,81,82,89,101,102, |
| Enlist social support | 92 |

**Table S4: Behaviour Changes Techniques to reduce excessive alcohol consumption**

| **Behaviour Changes Technique** | **Text messages** |
| --- | --- |
| 1. Provide information on consequences of excessive alcohol consumption and reducing excessive alcohol consumption | 7-11,15-18,30-35,37,38,87 |
| 2. Identify reasons for wanting and not wanting to reduce excessive alcohol consumption | 24-27,42-44,46-52,95,96 |
| 3. Boost motivation and self-efficacy | 36,39,50,52,59,69,71,75-77,84,87-88,90-93,95-96, 108 |
| 4. Provide normative information about others' behaviour and experiences | 5,6,13,14,23,26,27,37,38,68,99,100,109 |
| 11. Prompt use of imagery | 7,10,42,48,52,90,102 |
| 12. Model/demonstrate the behaviour | 22,23,25,26,40,47-51,50,55,57,58,62,63,65,66,68,  74,76,79,82,86,89,91,94,95,102,104,105,107-109 |
| 14. Facilitate goal setting | 53-58 |
| 15. Facilitate action planning/help identify relapse triggers | 60-66,70,72,74,77,83,101 |
| 16. Advise on avoidance of social cues for drinking | 72,80 |
| 17. Behaviour substitution | 79,85,86,89 |
| 18. Prompt review of goals | 68,74,77 |
| 19. Facilitate relapse prevention and coping | 70-77,80,83,93,95,101,105,108 |
| 20. Prompt self-recording | 12-14,18,19,21-23,45 |
| 21. Facilitate barrier identification and problem-solving | 72,73,74,77-82,95,101,105 |
| 23. Set graded tasks | 69 |
| 25. Change routine | 63-66,79,85,86,89 |
| 26. Advise on/facilitate use of social support | 40-44,63,67,79 |
| 28. Emphasize choice | 39,48,68 |
| 29. Assess current readiness and ability to reduce excessive alcohol consumption | 36,46,59,80,84,97 |
| 31. Assess current and past drinking behaviour | 4-6,9,12-14,21-23,30-36,70,97,108,109 |
| 32. Assess past history of attempts to reduce consumption | 5 |
| 36. Build general rapport | 1-3,17,20,28,29 |
| 38. Provide reassurance | 9,39,69,71,75,77,83,87,88,93,95,108 |
| 40. Elicit and answer questions | 12,21,24,30,36,42,46,49,52,56,59,64,73,80,84,85,90,97,98, 101, |
| 41. Elicit client views | 42,46,80,98, |

**Table S5: Mapping of the text messages on to the narrative**

| **Narrative** | **Text messages** | |
| --- | --- | --- |
|  | **Dave’s responses** | **Other characters’ responses/involvement** |
| Dave thinks he is a mature drinker | 5,13 |  |
| Through self-monitoring he realises he is actually a regular binge drinker | 11,17,18,22,23 | 6,14 |
| He identifies benefits of cutting down | 25 | 26,27 |
| Dave reports personal and social harms he has experienced | 31 | 32-35 |
| Dougie’s partner and child leave because of Dougie’s drinking |  | 37 |
| Dave’s wife is sympathetic which makes Dave realise he is also vulnerable | 38 |  |
| The importance of Alec’s support and other subjective norms is demonstrated | 40,41,43 | 44 |
| Dave reviews his reasons for wanting to cut down | 47,50 | 51 |
| Dave demonstrates self-efficacy in taking a decision to cut down | 55 |  |
| Dave models goal setting | 55 | 57,58 |
| He makes action plans | 62,63 | 65,66 |
| Demonstrates sticking to his goal/action plan | 68 |  |
| Demonstrates relapse | 70,72 |  |
| Action control | 95,96,105,108 | 81,82 |
| Coping planning | 74,78 | 102 |
| Recovery self-efficacy | 76 |  |
| Dave demonstrates substitutions for drinking sessions | 86,79 | 89 |
| Dave demonstrates benefits of sustained reduced drinking | 91,92,99 ,109 | 94,100 |
